# Chinese Parent Intention to Vaccinate Children With Special Diseases Against COVID-19

**DOI:** 10.3389/fpubh.2021.725980

**Published:** 2021-10-27

**Authors:** Xu Wang, Wu Yan, Lingling Lu, Liming Cao, Ye Tian, Kai Zhou

**Affiliations:** ^1^Department of Endocrinology, Children's Hospital of Nanjing Medical University, Nanjing, China; ^2^Department of Children Health Care, Children's Hospital of Nanjing Medical University, Nanjing, China; ^3^State Key Laboratory of Reproductive Medicine, School of Public Health, Nanjing Medical University, Nanjing, China; ^4^Key Laboratory of Modern Toxicology of Ministry of Education, School of Public Health, Nanjing Medical University, Nanjing, China; ^5^Department of Infection, Children's Hospital of Nanjing Medical University, Nanjing, China

**Keywords:** COVID-19, vaccine, children, special diseases, China

## Abstract

**Background:** Information on the intention of parents of children with special diseases to vaccinate their children against coronavirus disease 2019 (COVID-19) is scarce.

**Methods:** In this survey, all participants (*n* = 914) were enrolled from a tertiary children's hospital between September 2020 and April 2021. A face-to-face questionnaire interview was conducted to collect information on the special diseases of children and parental attitudes about the COVID-19 vaccine. We compared the demographic and disease factors between the group of parents who were willing to vaccinate their children against COVID-19 and the group who were unwilling to vaccinate.

**Results:** Among 941 children, 58.1% (*n* = 547) were boys. The Mean age was 1.4 (SD 1.9) years. If the COVID-19 vaccine becomes available for the child, 470 (49.9%) of parents were willing to provide vaccination for their children. The less the education levels of the father or mother, the more likely they were to vaccinate their children (*P* = 0.003, *P* = 0.007). However, more intentions to vaccinate were provided in parents of children with COVID-19 prevention and control education (*P* < 0.001).

**Conclusion:** Our findings provided evidence that some parents are willing to vaccinate their children with special diseases against COVID-19. Professional knowledge about COVID-19 prevention and control may contribute to increased parental intention.

## Introduction

Coronavirus disease 2019 (COVID-19) is caused by SARS-CoV-2, and it has resulted in a global pandemic (1, 2). Vaccination is one of the most effective strategies to prevent and control the spread of COVID-19 and is also one of the most basic public health services provided by the government (3, 4). Given possible concerns about the safety and needs of the new vaccine, it is unclear whether people will overcome their skepticism about the COVID-19 vaccine. At this point, positive public opinion and public trust in the COVID-19 vaccine are critical to promoting widespread acceptance of the vaccine (5).

Currently, the COVID-19 vaccine is administered to adults aged 18 years in China (6). In terms of the development of the global pandemic, the proportion of cases in children showed an increasing trend (7). Although the symptoms in children are less severe than those in adults (8), studies have shown that the viral load in the nasopharynx in children is equal to or greater than that in adults (9). Children, who also need to attend school and other frequent gatherings, may play an important role in the spread of the virus. At present, clinical trials of the COVID-19 vaccine in children are underway, but there is still insufficient data on children vaccinated with COVID-19 (10). Understanding the intention of parents to vaccinate children and the influence factors can help to achieve wider acceptance of the vaccine, leading to higher levels of immunity in the population. Among children, there is a special group with a high risk of infection, which involves several special disease states such as tumors, hematological system diseases, nervous system diseases, heart diseases, newborns, and so on. Such special populations might also be taken into account when developing future strategies to cover children with vaccination.

The timing for the availability of a safe and effective COVID-19 vaccine for children is uncertain, but it is important to anticipate and reduce barriers to its widespread use. To be more scientific and to provide vaccination for children with special diseases in the future, we evaluated the COVID-19 vaccine intentions of 941 cases in typical parents of children with special diseases. In addition, in the clinical real world, these parents were randomly assigned to two different pediatricians to assess their COVID-19 vaccine intentions with or without COVID-19 prevention and control education. We explored the differences in the intention of these parents with or without this education.

## Materials and Methods

### Study Design and Participants

Our study included children with special diseases who attended the Children's Hospital of Nanjing Medical University between September 2020 and April 2021, and their parents agreed to participate in this investigation, so one of the parents was invited for an investigation of intention to vaccinate their children against COVID-19. A face-to-face questionnaire interview was conducted to collect demographic characteristics, information on the special diseases of children, and attitudes about the COVID-19 vaccine.

We asked parents to answer these questions: “There is currently no COVID-19 vaccine for children. If there was a COVID-19 vaccination available in the future, would you give it to your child?” to obtain information about their intention to vaccinate. Besides, “what would you rate your child's vaccination risk score?” with a score between 0 (no risk at all) and 100 (highest risk).

In addition, children's age, gender, height, weight, preterm birth, cesarean delivery, food allergy, drug allergy, vaccination allergy, passive smoking, paternal education level, maternal education level, and special diseases of children were collected from questionnaire interview. The study was approved by the Medical Ethics Committee of Children's Hospital of Nanjing Medical University. We obtained signed informed consent from the parents of their children. Data used in this work were anonymous, and no individually identifiable information was available here.

### Statistical Analysis

There were 25 special diseases in children, namely, jaundice, congenital heart disease, febrile convulsion, epilepsy, preterm birth, cerebral palsy, intracranial bleeding, crissum abscess, etc. Data are presented as mean ± SD or frequency (%). To compare demographic and disease status between willing to vaccinate children against COVID-19 group and unwilling group, the chi-squared test was used for categorical variables and *t*-test for continuous variables. The level of significance was a two-sided *P* < 0.05. All analyses were performed in the SAS 9.2 (SAS Institute, Inc., Cary, NC, USA).

## Results

A total of 941 surveys were completed face to face. Of the 941 children, 788 children had one special disease, 118 children had two special diseases, 27 children had three special diseases, seven children had four special diseases, and one child had five special diseases. The proportion of congenital heart disease in children is the highest (18.8%) among all disease groups except for other unclassified diseases (Table 1).

**Table 1:** Overview of the special diseases in children.

**Disease**	**Frequency**	**Proportion (%)**
Jaundice	48	5.1
Congenital heart disease	177	18.8
Febrile convulsion	40	4.3
Epilepsy	32	3.4
Preterm birth	103	10.9
Cerebral palsy	11	1.2
Intracranial bleeding	43	4.6
Crissum abscess	61	6.5
Repeated infection	10	1.1
Asthma	7	0.7
Intravenous use of gamma globulin	25	2.7
Thrombocytopenic purpura	11	1.2
Eczema	31	3.3
Using hormones	6	0.6
Thrush	11	1.2
Respiratory infections, intestinal infections	14	1.5
Neutrophilic granulocytopenia or deficiency	59	6.3
Suspected vaccine allergy	46	4.9
Liver disease	17	1.8
Kidney disease	10	1.1
Allergic purpura	5	0.5
immunodeficiency	7	0.7
Before and after chemotherapy	3	0.3
Genetic metabolic diseases	7	0.7
Other diseases	354	37.6

*Seven hundred and eighty eight children with one special disease, One hundred and eighteen children with two special diseases, Twenty seven children with three special diseases, Seven children with four special diseases, and One child with five special diseases*.

Among 941 children, 58.1% (*n* = 547) were boys. The mean age was 1.4 (SD 1.9) years. The mean height was 74.2 (SD 18.9) cm, and the mean weight was 10.8 (SD 6.5) kg. A small number of children (10.9%) were preterm birth, and 40.6% were cesarean delivery. The majority have no history of allergies to food (87.7%), drugs (95.4%), or vaccines (94.0%). There were 35% (*n* = 329) of parents who confirmed passive smoke exposure of children. Most of the fathers (51.5%) or mothers (49.6%) had a bachelor's degree and above. If the COVID-19 vaccine becomes available for the child, the demographic characteristics of comparison between parents who were willing or unwilling to vaccinate their children against COVID-19 were showed in Table 2. A total of 470 (49.9%) of parents are willing to provide vaccination for their children and 471 (50.1%) of those are unwilling. The less the education levels of the father or mother, the more likely they are to vaccinate their children (*P* = 0.003, *P* = 0.007). Other basic characteristics did not differ between the willing and unwilling groups. Table 3 shows the distribution of parental self-assessment scores of children's vaccination risk, ranging between 0 and 100, and the 50th percentile is 41.

**Table 2:** Demographic characteristics of children with special diseases.

**Characteristics**		**Total (*n* = 941)**	**Willing (*n* = 470)**	**Unwilling (*n* = 471)**	***P-*values^**a**^**
Age	(year)	1.4 ± 1.9	1.4 ± 1.7	1.3 ± 2.0	0.740
Gender	Boy	547 (58.1)	277 (58.9)	270 (57.3)	0.616
Height	(cm)	74.2 ± 18.9	193 (41.1)	201 (42.7)	0.154
Weight	(kg)	10.8 ± 6.5	75.1 ± 18.4	73.3 ± 19.5	0.744
	Girl	394 (41.9)	10.9 ± 6.1	10.7 ± 6.9	
Preterm birth	No	838 (89.1)	418 (88.9)	420 (89.2)	0.908
	Yes	103 (10.9)	52 (11.1)	51 (10.2)	
Cesarean delivery	No	559 (59.4)	281 (59.8)	278 (59.0)	0.811
	Yes	382 (40.6)	189 (40.2)	193 (41.0)	
Food allergy	No	825 (87.7)	411 (87.4)	414 (87.9)	0.833
	Yes	116 (12.3)	59 (12.6)	57 (12.1)	
Drug allergy	No	898 (95.4)	444 (94.5)	454 (96.4)	0.158
	Yes	43 (4.6)	26 (5.5)	17 (3.6)	
Vaccination allergy	No	885 (94.0)	447 (95.1)	438 (93)	0.171
	Yes	56 (6.0)	23 (4.9)	33 (7)	
Passive smoking	No	612 (65.0)	450 (95.7)	459 (97.5)	0.255
	Yes	329 (35.0)	20 (4.3)	12 (2.5)	
Paternal education	Technical secondary school and below	225 (23.9)	133 (28.3)	92 (19.5)	**0.003**
	College	231 (24.5)	117 (24.9)	114 (24.2)	
	Bachelor degree and above	485 (51.5)	220 (46.8)	265 (56.3)	
Maternal education	Technical secondary school and below	248 (26.4)	144 (30.6)	104 (22.1)	**0.007**
	College	226 (24.0)	113 (24)	113 (24)	
	Bachelor degree and above	467 (49.6)	213 (45.3)	254 (53.9)	

a *Statistically significant results (P-values) are bolded*.

**Table 3:** Self-assessment score of vaccination in parents of children with specific diseases.

**Self-assessment score**	**Mean ± SD**	**Range**	**5th**	**25th**	**50th**	**75th**	**95th**
Risk score	39.2 ± 27.4	(0, 100)	0	17.5	41	52	99

Table 4 provides the comparison of the parental intention of children with special diseases to vaccinate their children against COVID-19. Greater willingness to vaccinate was showed in parents of children with congenital heart disease (*P* = 0.038). For other specific diseases, there was no difference between the willing and unwilling groups.

**Table 4:** Comparison of the intention of parents of children with special diseases to vaccinate their child against COVID-19.

	**Willing (*n* = 470)**	**Unwilling (*n* = 471)**	***P-*values^**a**^**
Jaundice	450 (95.7)	443 (94.1)	0.239
	20 (4.3)	28 (5.9)	
Congenital heart disease	394 (83.8)	370 (78.6)	**0.038**
	76 (16.2)	101 (21.4)	
Febrile convulsion	447 (95.1)	454 (96.4)	0.329
	23 (4.9)	17 (3.6)	
Epilepsy	454 (96.6)	455 (96.6)	0.995
	16 (3.4)	16 (3.4)	
Preterm birth	418 (88.9)	420 (89.2)	0.908
	52 (11.1)	51 (10.8)	
Cerebral palsy	466 (99.1)	464 (98.5)	0.365
	4 (0.9)	7 (1.5)	
Intracranial bleeding	452 (96.2)	446 (94.7)	0.278
	18 (3.8)	25 (5.3)	
Crissum abscess	439 (93.4)	441 (93.6)	0.888
	31 (6.6)	30 (6.4)	
Repeated infection	466 (99.1)	465 (98.7)	0.753
	4 (0.9)	6 (1.3)	
Asthma	467 (99.4)	467 (99.2)	1
	3 (0.6)	4 (0.8)	
Intravenous use of gamma globulin	459 (97.7)	457 (97)	0.547
	11 (2.3)	14 (3)	
Thrombocytopenic purpura	467 (99.4)	463 (98.3)	0.13
	3 (0.6)	8 (1.7)	
Eczema	452 (96.2)	458 (97.2)	0.358
	18 (3.8)	13 (2.8)	
Using hormones	467 (99.4)	468 (99.4)	1
	3 (0.6)	3 (0.6)	
Thrush	463 (98.5)	467 (99.2)	0.361
	7 (1.5)	4 (0.8)	
Respiratory infections, intestinal infections	464 (98.7)	463 (98.3)	0.593
	6 (1.3)	8 (1.7)	
Neutrophilic	437 (93)	445 (94.5)	0.342
granulocytopenia or deficiency	33 (7)	26 (5.5)	
Suspected vaccine allergy	451 (96)	444 (94.3)	0.229
	19 (4)	27 (5.7)	
Liver disease	461 (98.1)	463 (98.3)	0.803
	9 (1.9)	8 (1.7)	
Kidney disease	465 (98.9)	466 (98.9)	1
	5 (1.1)	5 (1.1)	
Allergic purpura	466 (99.1)	470 (99.8)	0.369
	4 (0.9)	1 (0.2)	
Immunodeficiency	467 (99.4)	467 (99.2)	1
	3 (0.6)	4 (0.8)	
Before and after chemotherapy	468 (99.6)	470 (99.8)	0.999
	2 (0.4)	1 (0.2)	
Organ transplantation or stem cell transplantation	470 (100)	471 (100)	–
Genetic metabolic diseases	466 (99.1)	468 (99.4)	0.998
	4 (0.9)	3 (0.6)	
Other diseases	285 (60.6)	302 (64.1)	0.27
	185 (39.4)	169 (35.9)	

a *Statistically significant results (P-values) are bolded*.

Table 5 shows the comparison of the intention of parents of children with special diseases to vaccinate their children against COVID-19 with or without COVID-19 prevention and control education. During the survey, these parents were randomly assigned to two different pediatricians to assess their COVID-19 vaccine intentions. Pediatrician A promoted professional knowledge about the prevention and control of COVID-19 for parents, while pediatrician B conducted a direct survey without a professional knowledge education. More intentions to vaccinate were provided in parents of children with COVID-19 prevention and control education (*P* < 0.001).

**Table 5:** Comparison of the intention of parents of children with special diseases to vaccinate their child against COVID-19 with or without COVID-19 education.

**Characteristics**		**Total** **(*n* = 941)**	**Willing** **(*n* = 470)**	**Unwilling** **(*n* = 471)**	***P-*values^**a**^**
COVID-19 education	No	280	32 (6.8)	248 (52.7)	**<0.001**
	Yes	661	438 (93.2)	223 (47.3)	

a *Statistically significant results (P-values) are bolded*.

## Discussion

Coronavirus disease 2019 (COVID-19) is highly infectious and the transmission process is complex. Children with special diseases are easy to be neglected, and once they are infected with the virus, it is easy to cause an outbreak. Vaccination of children with special diseases is necessary not only to prevent COVID-19 in them but also important for other general populations, including normal children and adults.

In our survey, overall, nearly one-half (50.1%) of participants indicated intentions to vaccinate their children with special diseases against COVID-19 when a vaccine for the child becomes available. In addition, more willingness to vaccinate was showed in parents of children with congenital heart diseases. For other specific diseases, there was no difference between the willing and unwilling groups. Previous studies have shown that parents of children with chronic conditions are less likely to vaccinate their children against COVID-19 (3). However, one study of other vaccines (such as the influenza vaccine) for children with chronic conditions suggested that children with chronic conditions were more likely to be vaccinated against influenza than children without chronic conditions (11). Our finding is especially striking considering that the survey was conducted with parents of children with special diseases, whose concerns about the safety of the vaccine, the state of diseases, and the stress of therapy may be more likely to influence their intentions to vaccinate their children against COVID-19.

To our surprise, we found that less parental educational attainment was a favorable factor in the willingness to vaccinate children against COVID-19, contrary to the findings of a national survey conducted by the prior reports. For example, higher education level was a factor associated with more willingness to vaccinate their children against A/H1N1 (12, 13). In the current study, concern about the safety of the COVID-19 vaccine may be one of the common reasons for uncertainty about vaccination. Therefore, we hypothesized that higher education might lead to more exposure to relevant information about vaccine risk reports, and therefore, more hesitation from parents to vaccinate their children.

However, our findings highlight the importance of professional COVID-19 prevention and control education. More intentions to vaccinate their children were provided in parents receiving this education. The spread of professional knowledge about COVID-19 may contribute to increased willingness to vaccinate. Furthermore, understanding why individuals may be hesitant to vaccinate their children may provide clues for developing policies to allay fears about vaccines (14, 15).

Taken together, these findings suggested that rational and accurate reporting of COVID-19 and vaccine characteristics in a way (e.g., through healthcare providers) that parents at all levels of education can understand may be an effective strategy for increasing vaccination of children in the future. Since children with special diseases are more vulnerable to infection than general children, 1 day, if there is a COVID-19 vaccine available for children, and children with special diseases are also applicable, special attention should be paid to this group of children.

The advantage of our study was that we focused on vaccination intentions of children with several special diseases, an understudied population group. Given the high prevalence of COVID-19, our finding might be meaningful to public health in the future. In addition, professional COVID-19 prevention and control education is a modifiable factor and may provide a clue to increased willingness to vaccinate. Although there was a relatively larger sample size of our survey about vaccination intentions of children with special diseases, the power of this single-center cohort study is still limited. Also, in our study, if more than one parent accompanied by a special disease in children, we only invited one of the parents to investigate, we asked the parents about their willingness to vaccinate children and their self-rating of the risk of vaccination for children. We did not further investigate the reasons for their reluctance to vaccinate their children.

In conclusion, in our survey, nearly one-half of the parents indicated intentions to vaccinate their children with special diseases against COVID-19 when a vaccine for the child becomes available, and the professional knowledge about COVID-19 may contribute to increased willingness to vaccinate.

## Data Availability Statement

The raw data supporting the conclusions of this article will be made available by the authors, without undue reservation.

## Ethics Statement

The studies involving human participants were reviewed and approved by Children's Hospital of Nanjing Medical University. Written informed consent to participate in this study was provided by the participants' legal guardian/next of kin.

## Author Contributions

XW: conception and design of the study. LL, LC, and YT: acquisition of data. WY: analysis and interpretation of data. XW and WY: drafting the article or revising it critically for important intellectual content. YT: epidemiological investigation guidance, verification of the full text, and payment of open access fee. XW and KZ: final approval of the version to be submitted. All authors contributed to the article and approved the submitted version.

## Conflict of Interest

The authors declare that the research was conducted in the absence of any commercial or financial relationships that could be construed as a potential conflict of interest.

## Publisher's Note

All claims expressed in this article are solely those of the authors and do not necessarily represent those of their affiliated organizations, or those of the publisher, the editors and the reviewers. Any product that may be evaluated in this article, or claim that may be made by its manufacturer, is not guaranteed or endorsed by the publisher.
